# Neuroprotective Effects and Mechanisms of Curcumin–Cu(II) and –Zn(II) Complexes Systems and Their Pharmacological Implications

**DOI:** 10.3390/nu10010028

**Published:** 2017-12-28

**Authors:** Fa-Shun Yan, Jian-Long Sun, Wen-Hai Xie, Liang Shen, Hong-Fang Ji

**Affiliations:** 1 Institute of Biomedical Research, Shandong University of Technology, Zibo 255000, Shandong, China; 18353365531@163.com (F.-S.Y.); 18369909086@163.com (J.-L.S.); xiewenhai@sdut.edu.cn (W.-H.X.); 2 Shandong Provincial Research Center for Bioinformatic Engineering and Technique, School of Life Sciences, Shandong University of Technology, Zibo 255000, Shandong, China

**Keywords:** curcumin, Alzheimer’s disease, metal ions, oxidative stress, PC12 cells

## Abstract

Alzheimer’s disease (AD) is the main form of dementia and has a steadily increasing prevalence. As both oxidative stress and metal homeostasis are involved in the pathogenesis of AD, it would be interesting to develop a dual function agent, targeting the two factors. Curcumin, a natural compound isolated from the rhizome of *Curcuma longa*, is an antioxidant and can also chelate metal ions. Whether the complexes of curcumin with metal ions possess neuroprotective effects has not been evaluated. Therefore, the present study was designed to investigate the protective effects of the complexes of curcumin with Cu(II) or Zn(II) on hydrogen peroxide (H_2_O_2_)-induced injury and the underlying molecular mechanisms. The use of rat pheochromocytoma (PC12) cells, a widely used neuronal cell model system, was adopted. It was revealed that curcumin–Cu(II) complexes systems possessed enhanced O_2_^·–^-scavenging activities compared to unchelated curcumin. In comparison with unchelated curcumin, the protective effects of curcumin–Cu(II) complexes systems were stronger than curcumin–Zn(II) system. Curcumin–Cu(II) or –Zn(II) complexes systems significantly enhanced the superoxide dismutase, catalase, and glutathione peroxidase activities and attenuated the increase of malondialdehyde levels and caspase-3 and caspase-9 activities, in a dose-dependent manner. The curcumin–Cu(II) complex system with a 2:1 ratio exhibited the most significant effect. Further mechanistic study demonstrated that curcumin–Cu(II) or –Zn(II) complexes systems inhibited cell apoptosis via downregulating the nuclear factor κB (NF-κB) pathway and upregulating Bcl-2/Bax pathway. In summary, the present study found that curcumin–Cu(II) or –Zn(II) complexes systems, especially the former, possess significant neuroprotective effects, which indicates the potential advantage of curcumin as a promising agent against AD and deserves further study.

## 1. Introduction

As the major form of dementia, Alzheimer’s disease (AD) is a progressive and irreversible neurodegenerative disease, which is related to aging and has a steadily increasing prevalence [1,2,3]. Due to the considerable burden on patients, family and society, screening efficient drugs for AD is a major challenge in drug discovery. Despite much effort being devoted to drug discovery of AD over the past decades, there is no effective therapeutic drug available to treat AD. The pathogenesis of AD remains far from being fully elucidated and oxidative damage to neurons caused by excessive reactive oxygen species (ROS) is widely recognized as an important factor [4,5,6,7]. Antioxidant supplement represents a promising strategy to combat AD. In addition, brain metal dysregulation, including copper (Cu(II)) and zinc (Zn(II)) ions, is intimately involved in the pathogenesis of AD [8,9,10]. The interaction of Cu(II) with both amyloid β (Aβ) peptides and the amyloid precursor protein (APP) has gained much attention in recent years [8,9,10]. Therefore, regulation of metal homeostasis is also considered to be a key therapeutic target. In recent years, many attempts have been made to explore metal chelators as potential agents against AD [11,12,13]. 

Curcumin is a yellow phenolic compound, isolated from the rhizome of turmeric (*Curcuma longa*), which has been intensively studied in the past decades owing to its wide spectrum of pharmacological activities, including antioxidant, anticancer, anti-inflammatory, and antimicrobial effects [14,15,16,17]. Curcumin can scavenge ROS and also chelate various metal ions [18,19,20]. Our previous studies found that the complexes of curcumin with metal ions possess superoxide dismutase (SOD)-like activity [21,22,23]. Thus, considering its abilities to scavenge free radicals and bind metal ions, curcumin may act as a promising agent to combat AD, especially in view of its good clinical safety, even at high dosage. In addition, it has been reported that curcumin can inhibit Aβ aggregation and fibril formation, promote the clearance of Aβ plaques, attenuate tau hyperphosphorylation, and inhibit acetylcholinesterase activity [24,25,26]. Rat pheochromocytoma (PC12) is a cell line derived from a pheochromocytoma of the rat adrenal medulla, which possesses the characteristics of neural cells and is widely used as a cell model system for neurodegenerative diseases [27,28,29,30,31]. With the aim of further exploring the neuroprotective effects of the metal ion complexes of curcumin and elucidating underlying mechanisms, we investigated the protective effects of curcumin and curcumin–Cu(II) or –Zn(II) complexes against hydrogen peroxide (H_2_O_2_)-induced injury in PC12 cells. Further mechanistic study demonstrated that curcumin–Cu(II) or –Zn(II) complexes inhibited cell apoptosis via downregulating the nuclear factor κB (NF-κB) and upregulating Bcl-2/Bax pathways.

## 2. Materials and Methods

### 2.1. Materials

Penicillin-streptomycin and Nutrient Mixture F-12 Ham were purchased from Sigma–Aldrich Shanghai Trading Co. (Shanghai, China). Horse serum (HS) was purchased from Solarbio Biological Technology Co., Ltd. (Beijing, China), fetal bovine serum (FBS) from Gibco (Glen Waverley, Australia). Curcumin, an extract from the turmeric powder of *Curcuma longa*, comprising the three main components—curcumin, demethoxycurcumin and bidemethoxycurcumin—was purchased from Shanghai Macklin Biological Technology Co., Ltd. (Shanghai, China). Copper chloride dehydrate, zinc chloride, pyrogallol, 1-(4,5-dimethylthiazol-2-yl)-3,5-diphenylformazan (MTT) and 2′,7′-dichlorofluorescindiacetate (DCFH-DA) were purchased from Sigma–Aldrich Shanghai Trading Co. Ltd. (Shanghai, China). H_2_O_2_ was purchased from Shuangshuang Chemical Co., Ltd. (Yantai, China). The kits for the Bradford protein assay, malondialdehyde (MDA), SOD, catalase (CAT) and glutathione peroxidase (GSH-Px) activities were gained from Nanjing Jiancheng Bioengineering Institute (Nanjing, China). Annexin V-fluorescein isothiocyanate (FITC)/propidium iodide (PI) apoptosis detection kit, caspase-3 and caspase-9 activity assay kits were purchased from Bibo Biological Technology Co., Ltd. (Nanjing, China). The enhanced chemiluminescence (ECL) kit, Bcl-2, Bax, p65 and β-actin antibody was purchased from Nanjing Enogene Biotech. Co., Ltd. (Nanjing, China). All other reagents were of analytical grade.

### 2.2. Superoxide Anion Radical Scavenging Assay

The superoxide anion radical (O_2_^·–^)-scavenging activities of curcumin and curcumin–Cu(II) or –Zn(II) complexes were measured with a modified pyrogallol autoxidation method [32]. Briefly, a pyrogallol solution (in 1 M HCl) was thoroughly mixed with Tris-HCl buffer at pH 7.4 and the absorption (A_325nm_) was recorded every 30 s for 5 min at 37 °C using a microplate reader (EVOLUTION 220, Thermo Fisher Scientific Oy, Vantaa, Finland). The O_2_^·–^-scavenging rate was estimated according to the following formula (T = 5 min):

(1) $$(\frac{\Delta A_{325\mathrm{nm},\mathrm{control}}}{T}-\frac{\Delta A_{325\mathrm{nm},\mathrm{sample}}}{T})/\frac{\Delta A_{325\mathrm{nm},\mathrm{control}}}{T}\times 100\%$$

The concentrations of curcumin and curcumin–Cu(II) or –Zn(II) complexes for 50% O_2_^·–^-scavenging was defined as the IC_50_ value.

### 2.3. Cell Culture and Viability Assay

PC12 cells, obtained from the Type Culture Collection of the Chinese Academy of Sciences (Shanghai, China), were cultured in Nutrient Mixture F-12 Ham medium, supplemented with 10% HS, 5% FBS, 100 U/ml of penicillin and streptomycin at 37 °C under 5% CO_2_. Before treatment, PC12 cells were seeded in 6 or 96-well plates (1 or 3 × 10^5^ cells/ml) and cultured for 24 h. Cells were pretreated with curcumin and curcumin–Cu(II) or –Zn(II) complexes for 0.5 h, before adding H_2_O_2_. Cell viability was assessed by an MTT assay [33]. After 24 h, the cells were incubated with H_2_O_2_ (500 µM) for an additional 24 h. Finally, 20 µL MTT was added to each well. After 4 h, 200 µL DMSO was added to each well to dissolve the formazan crystals. The absorbance was measured at 570 nm using a Multiwell microplate reader (MultiskanGo, Thermo Fisher Scientific Oy, Vantaa, Finland). Cell viability was expressed as the percentage of the control group.

### 2.4. ROS Assay

The intracellular ROS level was quantified using a DCFH-DA assay. At 24 h after seeding, the cells were pretreated with 25 µM curcumin and curcumin–Cu(II) or –Zn(II) complexes for 0.5 h and 500 µM H_2_O_2_ was added. After 6 h of incubation, the cells were washed with PBS (0.1 M, pH 7.4) and incubated with 10 µM DCFH-DA for 0.5 h in the dark. After treatment, the cells were washed with serum-free medium three times, and then pictures were taken with a fluorescence microscope (Olympus IX73, Tokyo, Japan), and relative fluorescence intensity was measured with a fluorescent microplate reader (Varioskan Flash, Thermo Fisher Scientific Oy, Vantaa, Finland). The intracellular ROS level in the control group was defined as 100% and the other groups were assessed as a percentage of the control.

### 2.5. Apoptosis Assay

Cell apoptosis was measured by annexin V-FITC/PI Staining. At 24 h after seeding, the cells were pretreated with curcumin and curcumin–Cu(II) or –Zn(II) systems for 0.5 h and H_2_O_2_ was added. After 6 h of incubation, the treated cells were washed with ice-cold PBS (0.1 M, pH 7.4) twice. Cells were resuspended in mixture of 500 µL of 1× binding buffer, 5 µL annexin V-FITC and PI for 10 min in the dark, and then analyzed by a fluorescence microscope (Olympus IX73, Tokyo, Japan).

### 2.6. MDA and Antioxidant Enzymes Assay

Treatment with curcumin or curcumin–Cu(II) or –Zn(II) complexes on PC12 cells was same as the above assay. After 6 h of incubation, the treated cells were washed twice with ice-cold PBS (0.1 M, pH 7.4) and homogenized. The homogenate was centrifuged at 12,000 rpm for 15 min at 4 °C, and then supernatant was collected for further experiments. The MDA content was evaluated using the 2-thiobarbituric acid assay [34]. Protein content was measured by the Bradford method [35]. The levels of intracellular antioxidant enzyme, CAT, total SOD and GSH-Px activities were determined according to the manuals of assay kits (Nanjing Jiancheng Bioengineering Institute, Nanjing, China).

### 2.7. Caspase-3 and Caspase-9 Activity

Treatment of PC12 cells with curcumin or curcumin–Cu(II) or–Zn(II) complexes was in accordance with the above assay. After 12 h of incubation, the treated cells were washed twice with ice-cold PBS and homogenized. The homogenate was centrifuged at 12,000 rpm for 15 min at 4 °C and then the supernatant of cell lysates was collected for further experiment. The activity of caspase-3 and caspase-9 were measured using assay kits (Nanjing Bibo Biological Technology, Nanjing, China).

### 2.8. Western Blotting

The cell lysates were centrifuged at 12,000 rpm for 15 min at 4 °C and then protein was separated by sodium dodecyl sulfate-polyacrylamide gel electrophoresis and transferred to PVDF membranes, which were blocked in 5% skim milk powder for 1 h, and then incubated with primary antibodies overnight at 4 °C. On the second day, the membrane was washed by TBST thrice, and then incubated with the secondary antibody for 1.5 h. The membrane was developed in an ECL reagent and visualized by a chemiluminescence detection system (ChemiScope 5300, Clinx, Shanghai, China).

### 2.9. Statistical Analysis

All independent experiments were conducted in triplicate. The data were shown as the mean ± standard deviation (SD). Statistical analysis was performed using SPSS (16.0) software (IBM Corporation, Armonk, NY, USA). The difference in the different groups was analyzed by one-way analysis of variance (ANOVA) and Tukey’s multiple comparisons *post-hoc* test. The significance levels were defined as ^#^ *p* < 0.01, ** *p* < 0.01 and * *p* < 0.05.

## 3. Results

### 3.1. O_2_^·–^-Scavenging Activities

O_2_^·–^-scavenging activities of curcumin and curcumin–Cu(II) or –Zn(II) complexes were examined and the results are shown in Table 1. It was found that curcumin–Cu(II) complexes exhibited enhanced O_2_^·–^-scavenging activities significantly more than parent curcumin and curcumin–Zn(II) complexes.

### 3.2. Cell Viability

**Figure 1:**
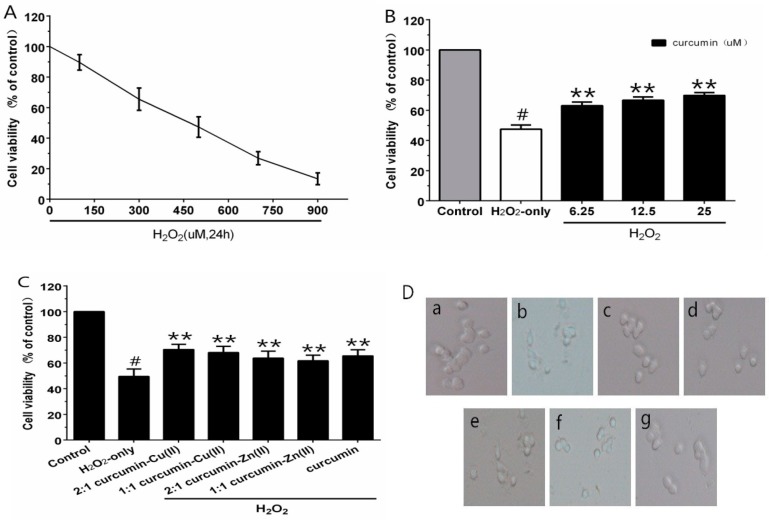
Curcumin and the complexes systems protected PC12 cells against H_2_O_2_-induced damage. Cytotoxic effects of H_2_O_2_ (**A**) on PC12 cells and the protective effects of curcumin (**B**) and the complexes (**C**) on H_2_O_2_-induced cytotoxicity to PC12 cells and morphological alteration (**D**). (**a**) control cells; (**b**) cells treated with H_2_O_2_ only; (**c**) cells pretreated with the 2:1 curcumin–Cu(II) complex system and co-treated with H_2_O_2_; (**d**) cells pretreated with the 1:1 curcumin–Cu(II) complex system and co-treated with H_2_O_2_; (**e**) cells pretreated with the 2:1 curcumin–Zn(II) complex system and co-treated with H_2_O_2_; (**f**) cells pretreated with the 1:1 curcumin-Zn(II) complexsystem and co-treated with H_2_O_2_; (**g**) cells pretreated with curcumin and co-treated with H_2_O_2_. (^#^ *p* < 0.01 versus control; * *p* < 0.05 and ** *p* < 0.01 versus H_2_O_2_-treated cells).

Different concentrations of H_2_O_2_ were employed for cytotoxic tests, to build the oxidative injury model of PC12 cells. As estimated by the MTT assay, cell viability was significantly decreased to 47.3% after treatment with 500 µM H_2_O_2_ for 24 h (Figure 1A). Thus, 500 μM H_2_O_2_ was regarded as the oxidative injury model for the subsequent experiments. When cells were pre-incubated with curcumin (6.25–25 µM) for 0.5 h, as shown in Figure 1B, curcumin (25 µM) showed an efficient neuroprotective effect and the cell viability increased significantly by 22.4% compared with H_2_O_2_-treated cells. The cytotoxicity experiment indicated no obvious reduction in cell viability with the treatment of curcumin alone. Therefore, this concentration was used for further experiments. In the complex protection experiment, the curcumin–Cu(II) complex further increased cell viability in comparison with the curcumin group. However, the protective effects of the curcumin–Zn(II) complex was lower than curcumin (Figure 1C). It indicated that the curcumin–Cu(II) complex had relatively stronger protective effects than curcumin or the curcumin–Zn(II) complex, and the 2:1 curcumin–Cu(II) complex exhibited the most significant protective effect.

### 3.3. Intracellular ROS Levels

**Figure 2:**
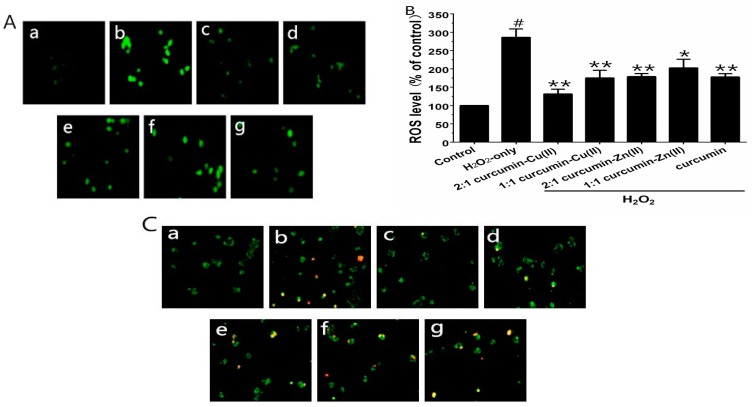
Inhibitory effects of curcumin and the complexes systems on the H_2_O_2_-induced reactive oxygen species (ROS) level and apoptotis in PC12 cells. (**A**) Representative images of 1-(4,5-dimethylthiazol-2-yl)-3,5-diphenylformazan (MTT) and 2′,7′-dichlorofluorescindiacetate (DCFH-DA) staining in PC12 cells by fluorescent microscopy (×100); (**B**) Intracellular reactive oxygen species (ROS) level; (**C**) Representative images of AV-FITC/PI staining under the microscope (×100). (**a**) control cells; (**b**) cells treated with H_2_O_2_ only; (**c**) cells pretreated with the 2:1 curcumin–Cu(II) complex system and co-treated with H_2_O_2_; (**d**) cells pretreated with the 1:1 curcumin–Cu(II) complex system and co-treated with H_2_O_2_; (**e**) cells pretreated with the 2:1 curcumin-­Zn(II) complex and co-treated with H_2_O_2_; (**f**) cells pretreated with the 1:1 curcumin-­Zn(II) complex system and co-treated with H_2_O_2_; (**g**) cells pretreated with curcumin and co-treated with H_2_O_2_. (^#^ *p* < 0.01 versus control; * *p* < 0.05 and ** *p* < 0.01 versus H_2_O_2_-treated cells).

To verify whether curcumin and curcumin–Cu(II) or –Zn(II) complexes systems could prevent H_2_O_2_-induced ROS generation, the ROS levels were measured using the fluorescence probe DCFH-DA. Exposure of the cells to 500 µM H_2_O_2_ for 6 h significantly increased the intracellular ROS level, in comparison with control group (Figure 2A). PC12 cells pretreated with curcumin and curcumin–Cu(II) or –Zn(II) complexes system significantly reduced ROS levels. The 2:1 curcumin–Cu(II) complex exhibited more significant effects than curcumin and other complexes (Figure 2B). To further verify whether H_2_O_2_-induced cell death was via cell apoptosis, AV/PI staining was used to detect apoptotic cells. After treatment with H_2_O_2_, the nuclei of most PC12 cells were stained, indicating that cells were apoptotic (Figure 2C). The treatment of curcumin and curcumin–Cu(II) or –Zn(II) complexes systems on H_2_O_2_-treated PC12 cells displayed significantly reduced numbers of stained cells (Figure 2C), indicating that both curcumin and curcumin–Cu(II) or –Zn(II) complexes systems could attenuate cell apoptosis induced by H_2_O_2_.

### 3.4. MDA Levels and Antioxidant Enzyme Activities

**Figure 3:**
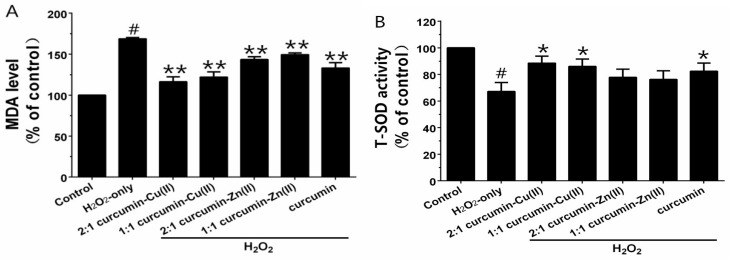

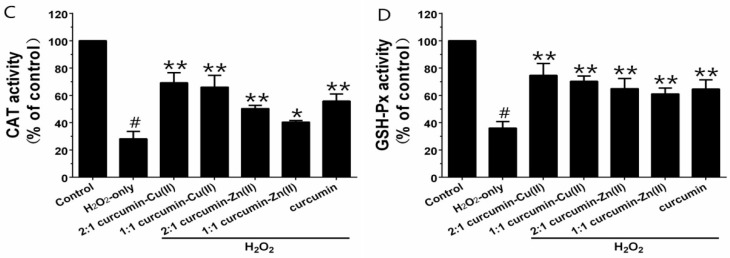
Effects of curcumin and the complexes on the malondialdehyde (MDA) level (**A**) and the activities of total superoxide dismutase (SOD) (**B**); catalase (CAT) (**C**) and glutathione peroxidase (GSH-Px) (**D**) in PC12 cells. PC12 cells were pretreated with curcumin and the complexes for 0.5 h before being exposed to 500 µM H_2_O_2_ for 6 h. (^#^ *p* < 0.01 versus control; * *p* < 0.05 and ** *p* < 0.01 versus H_2_O_2_-treated cells).

Exposure of PC12 cells to H_2_O_2_ caused an increase in the intracellular MDA level, and pretreatment with curcumin and curcumin–Cu(II) or –Zn(II) complexes systems obviously attenuated this increase (Figure 3A). Additionally, exposure of PC12 cells to H_2_O_2_ caused a decrease in the activities of SOD, CAT and GSH-Px (Figure 3B-D). Pretreatment with curcumin and curcumin–Cu(II) or –Zn(II) complexes systems significantly attenuated the changes in CAT, SOD and GSH-Px activities induced by H_2_O_2,_ and the protective effects of the curcumin–Cu(II) complex system were stronger than curcumin or the curcumin–Zn(II) complex system. 

### 3.5. Caspase-3 and Caspase-9 Activities

**Figure 4:**
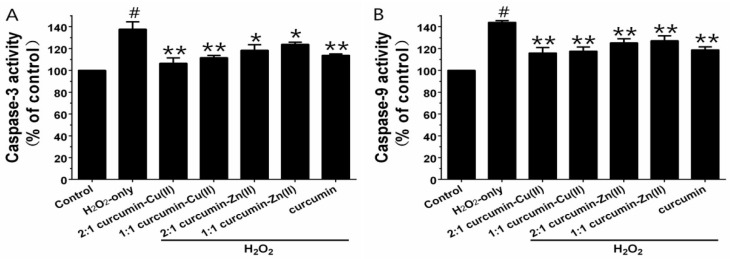
Effects of curcumin and the complexes systems on caspase-3 (**A**) and -9 (**B**) activity in H_2_O_2_-induced PC12 cells. Cells were pretreated with curcumin and the complexes systems for 0.5 h before being exposed to 500 µM H_2_O_2_ for 6 h. Caspase-3 and -9 activities were determined using a commercial kit, in accordance with the instructions of the manufacturer. (^#^ *p* < 0.01 versus control; * *p* < 0.05 and ** *p* < 0.01 versus H_2_O_2_-treated cells).

Caspase-3 and -9 are the key executive proteins related to apoptosis. As shown in Figure 4, H_2_O_2_ obviously increased caspase-3 and -9 activities and pretreatment with curcumin and curcumin–Cu(II) or –Zn(II) complexes attenuated the increases. The effect of the 2:1 curcumin–Cu(II) complex was the most significant—it decreased caspase-3 activity from 137.63 ± 6.8% to 106.37 ± 5.05% and caspase-9 activity from 143.93 ± 1.56% to 115.83 ± 5.08%.

### 3.6. Bcl-2/Bax Ratio and NF-κB p65 Levels

**Figure 5:**
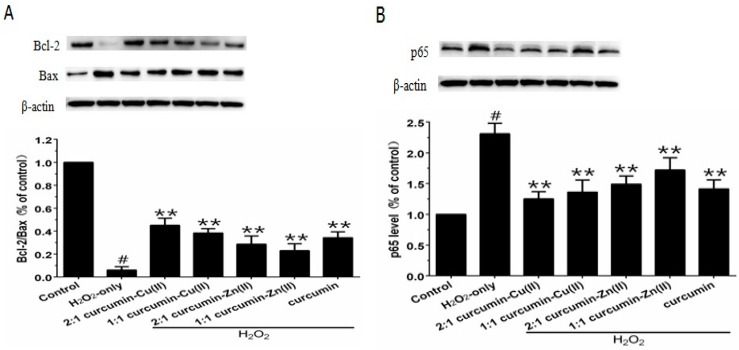
Effects of curcumin and the complexes system on the ratios of Bcl-2/Bax and p65 levels in PC12 cells. The PC12 cells were pretreated with curcumin and the complexes for 0.5 h and then exposed to 500 μM H_2_O_2_ for 12 h. (**A**) The expression of Bcl-2/Bax, β-actin was used for normalization and verification of protein loading, Bcl-2 and Bax in control, H_2_O_2_, curcumin and the complexes systems treatments and β-actin representations of the ratios of Bcl-2/Bax; (**B**) Following the same treatment, the p65 levels were identified. Data are shown as mean ± SD (*n* = 3). (^#^ *p* < 0.01 versus control; ** *p* < 0.01 versus H_2_O_2_-treated cells).

The Bcl-2/Bax ratio is an important factor in most mitochondria apoptotic proceedings. As shown in Figure 5A, exposing PC12 cells to H_2_O_2_ caused a significant decrease in the Bcl-2/Bax ratio, which was reversed by pretreatment with curcumin and curcumin–Cu(II) or –Zn(II) complexes systems. NF-κB is one of the major anti-apoptotic factors induced by ROS [29]. NF-κB is also regulated by various apoptotic stimuli or inhibitors. As shown in Figure 5B, Western blot results showed that p65 expression was increased in the presence of H_2_O_2_, while pretreatment with curcumin and curcumin–Cu(II) or –Zn(II) complexes systems significantly decreased the expression of p65 induced by H_2_O_2_. The effect of the 2:1 curcumin–Cu(II) complex system was stronger than curcumin or the other complexes.

## 4. Discussion

Screening multi-function drugs from natural products to treat AD has gained much interest in recent years [26,36,37]. Curcumin is an intensively investigated natural polyphenol with antioxidant activity, which can scavenge various ROS and reactive nitrogen species (RNS), and inhibit lipid peroxidation [19]. Curcumin has been found to be a metal ion chelator and can bind various metal ions. It is interesting to explore whether metal ion complexes of curcumin possess neuroprotective effects, which will endow curcumin another advantage against AD. The present study revealed that curcumin can inhibit H_2_O_2_-induced injury in neuronal PC12 cells, which is consistent with previous studies [29,38,39]. The effective concentration of curcumin employed in the present study was lower than that in the study of Siddiqui et al., which may arise from the different concentrations of employed H_2_O_2_ and a dose-dependent vulnerability of PC12 cells against H_2_O_2_ exposure [39]. Both curcumin–Cu(II) and –Zn(II) complexes were found to possess neuroprotective effects on PC12 cells, and the protective effects of the curcumin–Cu(II) complexes were relatively stronger than parent curcumin. The significant neuroprotective effects of curcumin–Cu(II) complexes may arise from the SOD-like activities of curcumin–Cu(II) complexes [21,22,23]. Curcumin–Cu(II) and –Zn(II) complexes significantly increased the activities of antioxidant enzymes, including SOD, CAT and GSH, and attenuated the H_2_O_2_-induced increase in MDA levels and caspase-3 and caspase-9 activities in PC12 cells. Previous studies have reported that many genes are involved in cell apoptosis, such as Bcl-2 and Bax genes [40]. In our study, pretreatment with curcumin and curcumin–Cu(II) or -Zn(II) complexes upregulated the expression of anti-apoptotic protein Bcl-2, and down-regulated the expression of pro-apoptotic protein Bax, indicating that curcumin and curcumin-Cu(II) or -Zn(II) complexes systems inhibited cell apoptosis, via upregulating the Bcl-2/Bax pathway. NF-κB acts as an important anti-apoptotic factor and it was demonstrated that the link between oxidative stress and NF-κB mainly arises from the inhibition of NF-κB activation by many antioxidants [41,42]. Our study indicated that curcumin and curcumin–Cu(II) or –Zn(II) complexes inhibit cell apoptosis via downregulating the NF-κB pathway, which is consistent with previous studies [28,43]. However, curcumin was reported to have an extremely poor bioavailability, which makes its pharmacology difficult to be fully elucidated and also hampers its clinical applications. In view of its low stability [44,45], our previous studies proposed that the bioactive degradation products of curcumin should contribute to in its diverse pharmacological effects [25,46,47]. The degradation products retain the main functional groups of curucmin and exhibit similar biological activities and mechanisms as parent curcumin to combat AD, including antioxidants, Aβ fibril formation-, and enzyme-inhibiting activities [25,46]. Additionally, many strategies have been used to improve the bioavailability of curcumin [48]. For instance, it was found that the application of adjuvants, like piperine, can significantly improve the bioavailability of curcumin [49].

## 5. Conclusions

To summarize, the present findings suggest that metal complexes of curcumin, especially curcumin–Cu(II), exhibit strong neuroprotective effects on neuronal PC12 cells. In view of the antioxidant and metal ion-chelating activities of curcumin, the neuroprotective effect of its metal complexes indicates the great advantages of curcumin as a promising anti-AD agent. In addition, it has been demonstrated that curcumin could inhibit Aβ aggregation and ameliorate Aβ-induced toxicity [25,50,51,52], and inhibit acetylcholinesterase activity [25,53]. Thus, the potentials of curcumin as a natural agent to combat AD deserve more preclinical and clinical studies.

## Figures and Tables

**Table 1:** Superoxide anion radical-scavenging activities of curcumin and the complexes. Data were expressed as the mean ± standard deviation (SD), *n* = 3. (* *p* < 0.05 and ** *p* < 0.01 versus curcumin).

Compounds	IC_50_ (µM)
2:1 curcumin–Cu(II) complex	238.14 ± 15.83 **
1:1 curcumin–Cu(II) complex	171.86 ± 14.86 **
2:1 curcumin–Zn(II) complex	323.49 ± 17.31
1:1 curcumin–Zn(II) complex	357.85 ± 12.93 *
curcumin	307.89 ± 15.42
